# Molecular diagnosis of bovine genital campylobacteriosis using high-resolution melting analysis

**DOI:** 10.3389/fmicb.2022.969825

**Published:** 2022-09-09

**Authors:** Marta Filipa Silva, Sabine Kienesberger, Gonçalo Pereira, Luísa Mateus, Luís Lopes-da-Costa, Elisabete Silva

**Affiliations:** ^1^Faculdade de Medicina Veterinária, Centro de Investigação Interdisciplinar em Sanidade Animal (CIISA), Universidade de Lisboa, Lisbon, Portugal; ^2^Associate Laboratory for Animal and Veterinary Science (AL4AnimalS), Lisbon, Portugal; ^3^Institute of Molecular Biosciences, University of Graz, Graz, Austria; ^4^BioTechMed-Graz, Graz, Austria; ^5^Field of Excellence BioHealth, University of Graz, Graz, Austria

**Keywords:** *Campylobacter fetus* subsp. *venerealis*, *Campylobacter fetus* subsp. *fetus*, bovine genital campylobacteriosis, real-time PCR, high-resolution melting

## Abstract

Bovine Genital Campylobacteriosis (BGC) is a worldwide spread venereal disease of cattle caused by *Campylobacter fetus* subsp. *venerealis* (*Cfv*). Although several real-time PCR assays were developed for *Cfv* identification, most target mobile genetic elements, which may lead to false-positive diagnosis. In this study, a real-time PCR assay coupled with High-Resolution Melting analysis (HRM) was developed for the identification of *Campylobacter fetus* subspecies and application in BGC diagnosis. Two HRM assays targeting different single nucleotide polymorphisms were validated using 51 *C. fetus* strains, including 36 *Cfv* and 15 *C. fetus* subsp. *fetus* (*Cff*). The specificity was assessed in 50 preputial samples previously tested as negative for *C. fetus* and in 24 strains from other *Campylobacter* species. The analytical sensitivity was determined with ten-fold dilutions of *Cfv* genome copies and in preputial samples spiked with *Cfv* cells. Both HRM assays accurately identified the 51 *C. fetus* strains, showing 100% concordance with the previous identification. *C. fetus* subspecies identification by HRM showed concordant results with the glycine test in 98.0% of the isolates. No amplification was obtained in *C. fetus* negative preputial samples as well as in strains from other *Campylobacter* species. The assays were able to detect 10^2^ genome copies of *Cfv*, while for preputial washing samples the limit of detection was 10^3^ CFU/mL. These novel HRM assays represent a highly specific and sensitive tool for the identification of *C. fetus* subspecies and show potential for direct use in bull preputial samples for BGC diagnosis.

## Introduction

Bovine Genital Campylobacteriosis (BGC) is a venereal bacterial disease of cattle caused by *Campylobacter fetus* subsp. *venerealis* (*Cfv*) (OIE, 2021). Bulls act as reservoirs of the disease by carrying *Cfv* in the genital tract for long periods of time (Silveira et al., 2018). Infection of females occurs during natural breeding or artificial insemination, and causes endometritis, embryonic mortality and abortion, resulting in cow infertility, poor herd reproductive performance, and economic losses to the cattle industry (Mshelia et al., 2010; Michi et al., 2016).

Diagnosis of BGC requires accurate identification of the causative agent, which is challenging due to the two *C. fetus* subspecies that can be present in cattle, *C. fetus* subsp. *fetus* (*Cff*) and *Cfv* (Silveira et al., 2018). These subspecies have highly syntenic genomes and exhibit similar phenotypic traits, hampering their differentiation by molecular methods or phenotypic assays (Sprenger et al., 2012; Silveira et al., 2018). Microbiological culture followed by phenotypic identification is the classic approach for *C. fetus* identification and subspecies differentiation, as recommended by the Organization for Animal Health (OIE) (OIE, 2021). This differentiation relies on the 1% glycine tolerance test, in which *Cfv* is intolerant, while *Cff* is tolerant to glycine (OIE, 2021). Nevertheless, diagnosis of BGC by microbiological culture is challenging due to the fastidious growth and poor survival of the pathogen (Mshelia et al., 2010). On the other hand, the polymerase chain reaction (PCR) has emerged as a promising technique to differentiate *C. fetus* subspecies with the advantage of not relying on bacterial viability (McMillen et al., 2006; Silveira et al., 2018). Several assays have been developed targeting differences in genomic features such as the *parA* gene and the insertion element ISCfe1 (McMillen et al., 2006; Abril et al., 2007; McGoldrick et al., 2013; van der Graaf-van Bloois et al., 2013). However, these targets can be transferred horizontally, which can lead to lack of specificity when used for diagnostic purposes in clinical samples (Spence et al., 2011; Silva et al., 2020a; Polo et al., 2021). Recently, *Cfv parA* and ISCfe1 homologs were detected in another inhabitant of the bovine genital tract, *Campylobacter portucalensis* (Silva et al., 2020b), identifying this microorganism as a cause of false-positive results in molecular *Cfv* detection assays (Silva et al., 2020a). These reports highlight the importance of developing alternative molecular assays for reliable detection and differentiation of *C. fetus* subspecies.

Previous studies have shown that some single nucleotide polymorphisms (SNPs) in the core-genome of *C. fetus* differentiate *Cff* from *Cfv* (Abdel-glil et al., 2020). In this context, real-time PCR followed by High-Resolution Melting (HRM) analysis would allow the detection of such variations in amplicon sequences. This method is based on the amplification of a target of interest in the presence of a dsDNA-binding dye, which exhibits high fluorescence in the bounded state to dsDNA and low fluorescence when unbonded. The high-resolution melting follows the amplification step, with the gradual denaturation of the amplicons due to small increments in the temperature, which originates a melting profile specific of each product (Chua et al., 2015). The equipment captures changes in the fluorescence signal with high precision at different temperature points, detecting accurately differences in the melting behavior of sequences differentiated by only one SNP (Life Technologies Corporation, 2010). In the last years, this method has been employed as a tool for the identification and differentiation of pathogens (Chua et al., 2015; Zhang et al., 2021; Ghorbani et al., 2022; Pakbin et al., 2022). In this study, we developed two HRM assays to detect SNPs that identify and differentiate the *C. fetus* subspecies. These assays have the potential to be applied directly in the analysis of clinical samples.

## Materials and methods

### *Campylobacter fetus* strains and culture conditions

Fifty-one *C. fetus* strains identified in previous studies as *Cfv* (*n* = 36) or *Cff* (*n* = 15; Supplementary Table 1), were used for the development of the HRM assays. Additionally, three *C. fetus* strains with non-consensual subspecies classification in previous studies were evaluated (Supplementary Table 1). Strains were grown on Columbia Blood Agar Plates, supplemented with 5% sheep blood (COS, Biomerieux, Marcy l’Étoile, France), at 37°C for 48 h under microaerophilic conditions (GenBox Microaer, Biomerieux, Marcy l’Étoile, France).

### Glycine tolerance test

Tolerance to 1% glycine was assessed following previously published recommendations (On and Holmes, 1991a,b). Briefly, plates were prepared by adding 1% glycine (Glycine molecular biology grade, AppliChem, Darmstadt, Germany) to Columbia agar (Columbia blood agar base, Hampshire, England) before autoclaving, and supplementing with 5% defibrinated sheep blood (Thermo Scientific, Hampshire, England) after cooling. After 48 h of growth, bacterial suspensions were prepared in phosphate-buffered saline (PBS) with a turbidity adjusted to 0.3 McFarland, using a Densimat densitometer (Biomerieux, Marcy-l’Étoile, France), corresponding to 10^8^ CFU/mL. Blood agar plates supplemented with 1% glycine were inoculated in triplicate with 20 μL drops of a bacterial suspension adjusted to 10^6^ CFU/mL, the spots allowed to dry, and incubated under microaerophilic conditions at 37°C for 72 h. To validate absence of bacterial growth on glycine plates, bacterial growth was confirmed on glycine-free plates. *Cfv* NCTC 10354 and *Cff* NCTC 10842 were used as negative and positive controls, respectively.

### DNA extraction

Genomic DNA of bacterial strains was isolated using DNeasy Blood and Tissue kit (Qiagen, Hilden, Germany) following manufacturer’s instructions. The purified DNA was quantified using a nanodrop 2000C spectrophotometer (Thermo Fisher Scientific, Waltham, MA, United States) and stored at −20°C until analysis.

### Real-time PCR-high-resolution melting analysis assays

#### Primer design

Three primer sets were designed to target three previously described SNPs (Abdel-glil et al., 2020), with potential to differentiate *Cfv* from *Cff*. The loci CFF8240_0641, CFF8240_1016, and CFF8240_1380 from the reference sequence of *Cff* 82-40 (NCBI accession no. CP000487.1) were selected for primer design, using Primer3web software v.4.1.0 (Koressaar and Remm, 2007; Untergasser et al., 2012; Kõressaar et al., 2018) and Primer Express software (Applied Biosystems, Foster City, United States), and Primer-BLAST (Ye et al., 2012) for *in silico* specificity confirmation. The primer-BLAST analysis included 18 *C. fetus* genomes and revealed an SNP in *Cff* 04/554 genome (Accession no.: CP008808.1) in the binding site of the reverse primer targeting CFF8240_1016. Although it was not possible to design a primer between this polymorphism and the targeted SNP due to their proximity, the assay was included in the study since among all sequenced genomes of *C. fetus* from bovines only strain *Cff* 04/554 displays this polymorphism.

**FIGURE 1:**
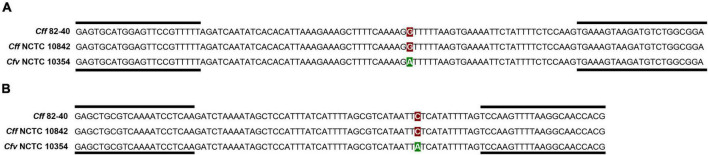
Schematic representation of the amplification products containing SNPs differentiating *Campylobacter fetus* subsp. *venerealis* from *C. fetus* subsp. *fetus.* The image shows the amplification products for loci CFF8240_0641 **(A)** and CFF8240_1016 **(B)** for strain *Cff* 82-40 and strains used as controls in this study, *Cff* NCTC 10842 and *Cfv* NCTC 10354. SNPs are highlighted in red (*Cff*) and green (*Cfv*); black lines represent primer binding sites.

A preliminary analysis revealed that primers targeting locus CFF8240_1380 produced non-specific amplification products in preputial samples negative for *C. fetus*, and were excluded from further analysis. The assays targeting loci CFF8240_1016 and CFF8240_0641, which encode a phosphatase from Ppx/GppA family and a Hit family protein, respectively, were selected for further analysis (Table 1 and Figure 1).

**TABLE 1:** Primer sequences used to identify and differentiate *C. fetus* subspecies.

Target	Primer sequence (5′–3′)	Amplicon size (bp)
CFF8240_0641	Fw: GAGTGCATGGAGTTCCGTTTTT	112
	Rv: TCCGCCAGACATCTTACTTTCA	
CFF8240_1016	Fw: GAGCTGCGTCAAAATCCTCAA	95
	Rv: CGTGGTTGCCTTAAAACTTGGA	

#### Real-time PCR-high-resolution melting analysis

Real time PCR assays were carried out in 20 μL reaction mixtures containing 1× MeltDoctor HRM Master Mix (pplied Biosystems, Foster City, United States), 0.3 μM of each primer, 2 ng of bacterial DNA or 1 μL of DNA from preputial samples. All samples were tested in triplicate and *C. fetus* strains were tested in three independent runs. *Cfv* NCTC 10354 and *Cff* NCTC 10842 were included as positive controls and used for variant call. The subspecies classification was based on the melting behavior of the controls included in each run. Amplification was performed on a 7500 FAST System (Applied Biosystems, Foster City, United States) using the following thermal conditions: an initialization step of 95°C for 10 mins, followed by 40 cycles of amplification with denaturation at 95°C for 15 s and annealing at 60°C for 1 min. The generated amplicons were then subjected to the HRM step, which was performed according to the manufacturer’s specifications: denaturation at 95°C for 10 s, annealing at 60°C for 1 min, followed by HRM up to 95°C for 15 s and annealing at 60°C for 15 s. The HRM analysis was performed using the High-Resolution Melt Software v3.0 (Applied Biosystems, Foster City, United States). A threshold cycle (Ct) < 35 was considered positive and the amplification products of eight representative *C. fetus* strains were sequenced (Stabvida, Almada, Portugal) to confirm the presence of the expected SNP.

#### Specificity and analytical sensitivity

The specificity of the assays was evaluated in 24 strains from other *Campylobacter* species (Supplementary Table 2), including *Campylobacter portucalensis* (*n* = 5), *Campylobacter sputorum* (*n* = 6), *Campylobacter lari* (*n* = 1), *Campylobacter lanienae* (*n* = 1), *Campylobacter coli* (*n* = 4), *Campylobacter jejuni* (*n* = 3), and *Campylobacter hyointestinalis* (*n* = 4). Additionally, a total of 50 preputial washing samples previously tested as negative for *C. fetus* by real time-PCR targeting the *nahE* gene (Silva et al., 2020a) were analyzed to evaluate the specificity of the HRM assay in clinical samples.

The analytical sensitivity was assessed by using 10-fold serial dilutions of DNA from *Cfv* strain NCTC 10354, as previously described (Silva et al., 2020a). Dilutions ranging from 1 × 10^1^ to 1 × 10^6^ genome copies were tested in triplicate, in three independent runs, to ensure reproducibility. The standard curve was analyzed for evaluation of linearity (*r*^2^), amplification efficiency (E) and reproducibility as previously described (Silva et al., 2020a).

Additionally, preputial samples from three bulls were spiked with *Cfv* strain NCTC 10354 to simulate positive samples. Briefly, bacterial cultures were suspended in PBS and adjusted to 0.3 McFarland (≈1 × 10^8^ CFU/mL), and suspensions diluted and added to preputial samples to attain final mixture concentrations ranging from 1 × 10^5^ to 1 × 10^1^ CFU/mL in 2 mL of preputial sample. DNA extraction was performed using 2 mL of sample, centrifuged at 5,000 × g for 10 min and the pellet was resuspended in 180 μL of buffer ATL (DNeasy Blood and Tissue kit, Qiagen, Hilden, Germany) for DNA isolation as described above for *C. fetus* isolates. The final step of elution was performed using 100 μL of buffer AE (DNeasy Blood and Tissue kit, Qiagen, Hilden, Germany).

#### Reproducibility

The intra- and inter-assay reproducibility were evaluated for all *C. fetus* strains, using the coefficient of variation (CV) of the melting temperature (Tm) value in three replicates tested on the same plate and in three independent runs, respectively.

#### Statistical analysis

Differences in the mean Tm between *Cfv* and *Cff* amplicons were evaluated with Student’s *t*-test using IBM SPSS Statistics 27.0 (IBM Corporation, Armonk, United States). Results of melting temperature are reported as mean of three independent runs ± standard deviation (SD). Values of *P* < 0.05 were considered statistically significant.

## Results

###  Classification of *Campylobacter fetus* strains

**FIGURE 2:**
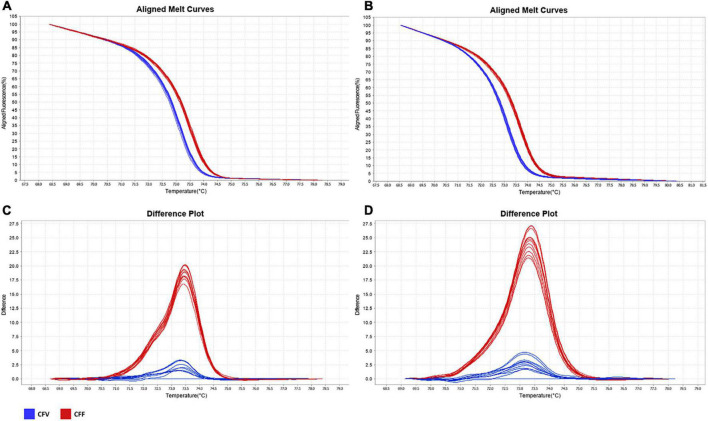
Melt curve analysis of *Campylobacter fetus* subsp. *venerealis* and *C. fetus* subsp. *fetus* amplification products. Aligned melt curves **(A,B)** and difference plots **(C,D)** of the assays targeting loci CFF8240_0641 **(A,C)** and CFF8240_1016 **(B,D)** obtained using High Resolution Melt Software. The difference plots were obtaining using as reference the curve of *Cfv* NCTC 10354. Blue curves: *C. fetus* subsp. *venerealis*; Red curves: *C. fetus* subsp. *fetus.*

The 51 *C. fetus* strains were evaluated by two real-time PCR assays directed to loci CFF8240_0641 and CFF8240_1016, followed by HRM analysis. Both real time PCR-HRM assays were able to segregate *C. fetus* strains in two distinct populations based on the Tm of the amplification products (Figure 2). The amplification of a single amplicon was confirmed by the presence of a single peak in each melt curve plot and by agarose gel electrophoresis. In addition, the differences in the Tm were confirmed to be associated with the expected SNPs by Sanger sequencing of five amplicons representative of both curve profiles. Both HRM assays identified *Cfv* and *Cff* isolates in agreement with the initial classification of the strains. All *Cfv* strains were sensitive to glycine, whereas *Cff* strains grew in glycine plates, with the exception of strain 98/v445.

The melting temperatures obtained for each isolate in the three independent runs are shown in Supplementary Table 3. The assay targeting CFF8240_0641 differentiated *Cfv* from *Cff* through a mean amplicon Tm of 73.34 and 73.74°C (*P* < 0.001), respectively (Table 2). The assay targeting CFF8240_1016 differentiated *Cfv* from *Cff* through a mean Tm of 73.11°C and 73.59°C (*P* < 0.001), respectively (Table 2). These assays showed low intra-assay coefficients of variation for all strains tested, which were less than or equal to 0.085 and 0.095% using primers for loci CFF8240_0641 and CFF8240_1016, respectively (Table 2), evidencing a good reproducibility between replicates. For both assays, strains were tested in different runs and the Tm results showed only minor differences across assays, as evidenced by the inter-assay CV less than or equal to 0.337 and 0.176% for CFF8240_0641 and CFF8240_1016, respectively (Table 2).

**TABLE 2:** Melting temperature in high-resolution melting assays to differentiate *Campylobacter fetus* subspecies.

Target	Mean Tm ± SD (°C)		Coefficient of variation (%)	
	*Cfv*	*Cff*	intra-assay	inter-assay
CFF8240_0641	73.34 ± 0.083^a^	73.74 ± 0.101^b^	≤ 0.085	≤ 0.337
CFF8240_1016	73.11 ± 0.106^a^	73.59 ± 0.056^b^	≤ 0.095	≤ 0.176

Results of the melting temperature are presented as mean melting temperature (Tm) ± standard deviation (SD). Different letters in the mean Tm ± SD indicate statistically significant differences (*P* < 0.001). *Cfv, C. fetus* subsp. *venerealis; Cff, C. fetus* subsp. *fetus.*

Three *C. fetus* strains (98/v444, BT 34/99, and 110800-21-2) with non-consensus subspecies classification in previous reports were also evaluated in this study. According to the glycine tolerance test and HRM assays, strains 98/v444 and BT 34/99 were here classified as *Cfv*, while strain 110800-21-2 was classified as *Cff* (Supplementary Table 3).

### Specificity and analytical sensitivity of the high-resolution melting analysis assays

The specificity of the assays was assessed by testing DNA from other *Campylobacter* species (Supplementary Table 2) and preputial washing samples previously classified as negative for *C. fetus*. No amplification and consequently no melting curves were obtained when using DNA of *C. portucalensis, C. sputorum, C. lari, C. lanienae, C. coli, C. jejuni*, and *C. hyointestinalis*. Both assays also produced negative results in the 50 preputial washing samples tested, which is consistent with the absence of amplification of the *nahE* gene, indicating the absence of non-specific amplification. Overall, both assays revealed 100% sensitivity and 100% specificity.

The analytical sensitivity of the assays was evaluated by ten-fold serial dilutions of genomic DNA of *Cfv* NCTC 10354. Results revealed that both real time PCR-HRM assays were able to detect 10^2^ genome copies with a cycle threshold (Ct) lower than 35 (Ct = 34.71 ± 0.12 for CFF8240_0641 and Ct = 34.60 ± 0.03 for CFF8240_1016) (Table 3). These results were reproducible in three independent runs, showing the same amplicon melting temperature. The standard curve revealed an amplification efficiency of 91.45 and 93.16% for CFF8240_0641 and CFF8240_1016 assays, respectively, with an *r*^2^ of 0.99 and coefficients of variation ≤ 1.7% (Table 4).

**TABLE 3:** Amplification results for *Campylobacter fetus* subsp. *venerealis* NCTC 10354 genome copies.

Genome copies/reaction	CFF8240_0641	CFF8240_1016
1000000	20.68 ± 0.05	20.79 ± 0.11
100000	23.95 ± 0.10	24.15 ± 0.05
10000	28.09 ± 0.25	27.92 ± 0.07
1000	31.34 ± 0.06	31.35 ± 0.06
100	34.71 ± 0.12	34.60 ± 0.03

Values are presented as the mean Ct of three runs ± standard deviation for assays targeting loci CFF8240_0641 and CFF8240_1016.

**TABLE 4:** Performance parameters of the real-time PCR assays.

Target	Slope	Y-intercept	*r* ^2^	*E* (%)	Intra-assay CV (%)	Inter-assay CV (%)
CFF8240_0641	−3.5454	41.937	0.99	91.45	≤1.70	≤1.08
CFF8240_1016	−3.4975	41.722	0.99	93.16	≤1.48	≤0.6

To evaluate the suitability of the assays for diagnosis in clinical samples, the limit of detection (LOD) was also assessed in preputial washing samples spiked with *Cfv*. The LOD of both assays was 10^3^ CFU/mL in three independent runs using preputial washing samples from three bulls. Amplification of preputial samples with 10^3^ CFU/mL occurred in thresholds cycles of 34.13 ± 0.23 and 33.86 ± 0.55 for assays targeting CFF8240_0641 and CFF8240_1016, respectively.

## Discussion

The accurate identification of *Cfv* is crucial for the diagnosis of BGC since only subspecies *venerealis* is recognized as the etiologic agent of the disease (OIE, 2021). Misidentification of subspecies *fetus* as *venerealis* originates considerable economic costs related to testing, culling, and control strategies such as artificial insemination. On the other hand, misidentification of a *Cfv* as *Cff* perpetuates the disease in the herd with the associated costs related to decreased reproductive efficiency. In the last years, several real-time PCR assays have been developed to detect subspecies venerealis-specific sequences, such as the insertion element ISCfe1, *parA*, and *virB11* genes (McMillen et al., 2006; McGoldrick et al., 2013; van der Graaf-van Bloois et al., 2013; Iraola et al., 2016). However, these sequences can be horizontally transferred and have been associated to specificity failures in real-time PCR assays (Spence et al., 2011; Silva et al., 2020a; Polo et al., 2021). Thus, accurate molecular diagnosis of BGC still requires the identification of molecular targets specific to *Cfv*.

A recent study based on whole-genome sequencing data identified SNPs differentiating ISCfe1 positive genomes, proposed as *Cfv*, from the remaining *C. fetus* strains (Abdel-glil et al., 2020). Real-time PCR coupled with HRM can differentiate SNPs and has emerged as a fast, easy to perform and cost-effective method for identification and differentiation of several bacterial pathogens (Zhang et al., 2021; Ghorbani et al., 2022; Pakbin et al., 2022).

In the present study, three of the SNPs proposed to differentiate the subspecies (Abdel-glil et al., 2020) were selected to develop real-time PCR assays coupled with HRM analysis to identify *C. fetus* subspecies. The most promising primer pairs target a Ppx/GppA family phosphatase (locus CFF8240_1016) and a Hit family protein (locus CFF8240_0641). Although these sequences differ by only one SNP, the melting behavior of the amplification products was significantly shifted, thus allowing subspecies differentiation. Both real-time PCR-HRM assays accurately identified the subspecies of 51 *C. fetus* strains, with unambiguously distinct melt curve profiles and melting temperature. Moreover, both assays revealed a good intra- and inter-assay reproducibility of the Tm values in all strains tested, evidenced by the low CV values. Although the Tm values showed slight differences between HRM runs, as observed in other studies (Naze et al., 2015; Ashrafi et al., 2017; Fehlberg et al., 2017), these differences were balanced by the inclusion of *Cfv* and *Cff* controls in each run. The subspecies classification was assigned based on the melting behavior of the *Cfv* and *Cff* controls included in each plate, whose inclusion is mandatory in all runs. We also evaluated strains with discrepant subspecies classification results in previous studies. Strains 98/v444 and BT 34/99 were here classified as *Cfv*, as indicated by Van Bergen et al. (2005), although they were typed as *Cff* in other studies (Wagenaar et al., 2001; Gorkiewicz et al., 2010). Strain 11800-21-2 was previously identified as *Cfv* (Gorkiewicz et al., 2010) but was in good agreement with other studies (Van Bergen et al., 2005; van der Graaf-Van Bloois et al., 2014, 2016a,b). The lack of standardized methods for subspecies classification and the absence of an explicit gold standard may be responsible for disagreeing classifications of *C. fetus* strains across studies. The developed HRM assays also have the potential to be implemented as an accurate method for the direct detection of *Cfv* in clinical samples. Both assays successfully detected *Cfv* in preputial samples spiked with 10^3^ CFU/mL. The suitability of the assays was also validated by the absence of non-specific amplification in preputial samples negative for *C. fetus*. Nevertheless, additional studies with samples from naturally infected animals and different matrixes, namely samples from aborted fetuses, should be tested to fully validate these assays for use in clinical samples. Additionally, the interlaboratory testing of these assays hereafter will be valuable to consider these assays as global diagnostic tools for the diagnosis of Bovine Genital Campylobacteriosis worldwide. Moreover, as we identified a polymorphism in the primer-binding site adjacent to the SNP in locus CFF8240_1016 in one bovine isolate (strain *Cff* 04/554), we cannot exclude specificity or sensitivity failures when other isolate collections or clinical samples are evaluated. This polymorphism may impact the amplification and/or melting temperature of the amplicons. In contrast, the assay targeting CFF8240_0641 proved to be effective without potential specificity issues, making it a preferential assay to be used for diagnosis.

This study also highlighted specificity failures of the glycine tolerance test, even when using standardized conditions such as inoculum size (10^6^ UFC/mL) and culture conditions. Although all *Cfv* were correctly identified by this phenotypic test, this would misidentify one *Cff* isolate. Previous studies already reported the occurrence of *Cfv* strains with tolerance to glycine (van der Graaf-Van Bloois et al., 2014, 2016a), which is acquired through mutation or transduction (Chang and Ogg, 1971), as well as *Cff* sensitivity to glycine (Wagenaar et al., 2001). Thus, this research also evidences the inconsistencies between the phenotypic analysis and the different molecular methods in the identification of *C. fetus* subspecies.

In conclusion, this study describes two real-time PCR-HRM assays for the highly specific and sensitive identification and differentiation of *Cfv* and *Cff*. Although exhibiting a similar performance in the present collection of strains, the assay targeting CFF8240_0641 is potentially more accurate due to possible, although presumably rare, polymorphisms in *Cff* strains. Importantly, the assays have the potential to be used for direct analysis of preputial samples and thus could prove to be a valuable tool for the diagnosis and control of BGC.

## Data availability statement

The original contributions presented in this study are included in the article/Supplementary material, further inquiries can be directed to the corresponding author.

## Ethics statement

Ethical review and approval was not required for the animal study because bovine preputial samples were collected by certified veterinarians using the OIE recommended sampling method as part of the breeding soundness examination of bulls and as a clinical service requested by owners to the Faculty of Veterinary Medicine of the University of Lisbon. As samples were collected for diagnostic purposes, according to EU and national legislation (Directive 2010/63/EU and Decree-law no. 113/2013), no ethical approval from an Institutional Animal Care and Use Committee or other relevant ethics board was required. According to the publicly available regulation of the Veterinary Teaching Hospital of the Faculty of Veterinary Medicine of the University of Lisbon, all clinical and diagnostic procedures and records may be used for teaching and research purposes while maintaining confidentiality. Written informed consent was obtained from the owners for the participation of their animals in this study.

## Author contributions

LL-D-C and ES: conceptualization, supervision, project administration, and funding acquisition. MS, LL-D-C, and ES: methodology and validation. MS, GP, LL-D-C, and ES: formal analysis. MS: investigation and writing—original draft preparation. SK, GP, LL-D-C, and ES: resources. SK, GP, LM, LL-D-C, and ES: writing—review and editing. MS, LM, LL-D-C, and ES: visualization. All authors have read and agreed to the published version of the manuscript.

## Supplementary material

Supplementary Table 1 *Campylobacter fetus* strains analyzed by real-time PCR followed by HRM analysis.

Supplementary Table 2 *Campylobacter* spp. strains used for specificity evaluation of real-time PCR-HRM assays.

Supplementary Table 3 Results of HRM analysis using primers targeting CFF8240_0641 and CFF8240_1016.

## References

[B1] Abdel-glil M. Y. Hotzel H. Tomaso H. Linde J. (2020). Phylogenomic analysis of *Campylobacter fetus* reveals a clonal structure of insertion element ISCfe1 positive genomes. *Front. Microbiol.* 11:585374. 10.3389/fmicb.2020.585374 33281781 PMC7688749

[B2] Abril C. Vilei E. M. Brodard I. Burnens A. Frey J. Miserez R. (2007). Discovery of insertion element IS Cfe1: A new tool for *Campylobacter fetus* subspecies differentiation. *Clin. Microbiol. Infect.* 13 993–1000. 10.1111/j.1469-0691.2007.01787.x 17697006

[B3] Ashrafi R. Bruneaux M. Sundberg L. R. Pulkkinen K. Ketola T. (2017). Application of high resolution melting assay (HRM) to study temperature-dependent intraspecific competition in a pathogenic bacterium. *Sci. Rep.* 7 1–8. 10.1038/s41598-017-01074-y 28428555 PMC5430548

[B4] Chang W. Ogg J. E. (1971). Transduction and mutation to glycine tolerance in vibrio fetus. *Am. J. Vet. Res.* 32 649–653. 4938782

[B5] Chua K. H. Lim S. C. Ng C. C. Lim Y. A. L. Lau T. P. Chai H. C. (2015). Development of high resolution melting analysis for the diagnosis of human malaria. *Sci. Rep.* 5:15671. 10.1038/srep15671 26507008 PMC4623528

[B6] Fehlberg H. F. Maciel B. M. Albuquerque G. R. (2017). Identification and discrimination of *Toxoplasma gondii, Sarcocystis* spp., *Neospora* spp., and *Cryptosporidium* spp. by righ-resolution melting analysis. *PLoS One* 12:e0174168. 10.1371/journal.pone.0174168 28346485 PMC5367704

[B7] Ghorbani J. Hashemi F. B. Jabalameli F. Emaneini M. (2022). Multiplex detection of five common respiratory pathogens from bronchoalveolar lavages using high resolution melting curve analysis. *BMC Microbiol.* 22:141. 10.1186/s12866-022-02558-2 35590256 PMC9118692

[B8] Gorkiewicz G. Kienesberger S. Schober C. Scheicher S. R. Gülly C. Zechner R. (2010). A genomic island defines subspecies-specific virulence features of the host-adapted pathogen *Campylobacter fetus* subsp. venerealis. *J. Bacteriol.* 192 502–517. 10.1128/JB.00803-09 19897645 PMC2805309

[B9] Iraola G. Pérez R. Betancor L. Marandino A. Morsella C. Méndez A. (2016). A novel real-time PCR assay for quantitative detection of *Campylobacter fetus* based on ribosomal sequences. *BMC Vet. Res.* 12:286. 10.1186/s12917-016-0913-3 27978826 PMC5159996

[B10] Koressaar T. Remm M. (2007). Enhancements and modifications of primer design program Primer3. *Bioinformatics* 23 1289–1291. 10.1093/bioinformatics/btm091 17379693

[B11] Kõressaar T. Lepamets M. Kaplinski L. Raime K. Andreson R. Remm M. (2018). Primer3-masker: Integrating masking of template sequence with primer design software. *Bioinformatics* 34 1937–1938. 10.1093/bioinformatics/bty036 29360956

[B12] Life Technologies Corporation (2010). *A guide to high resolution melting (HRM) analysis.* Carlsbad, CA: Life Tecnhologies Corporation.

[B13] McGoldrick A. Chanter J. Gale S. Parr J. Toszeghy M. Line K. (2013). Real Time PCR to detect and differentiate *Campylobacter fetus* subspecies fetus and *Campylobacter fetus* subspecies venerealis. *J. Microbiol. Methods* 94 199–204. 10.1016/j.mimet.2013.06.014 23811208

[B14] McMillen L. Fordyce G. Doogan V. J. Lew A. E. (2006). Comparison of culture and a novel 5′ Taq nuclease assay for direct detection of *Campylobacter fetus* subsp. Venerealis in clinical specimens from cattle. *J. Clin. Microbiol.* 44 938–945. 10.1128/JCM.44.3.938-945.2006 16517880 PMC1393111

[B15] Michi A. N. Favetto P. H. Kastelic J. Cobo E. R. (2016). A review of sexually transmitted bovine trichomoniasis and campylobacteriosis affecting cattle reproductive health. *Theriogenology* 85 781–791. 10.1016/j.theriogenology.2015.10.037 26679515

[B16] Mshelia G. D. Amin J. D. Woldehiwet Z. Murray R. D. Egwu G. O. (2010). Epidemiology of bovine venereal campylobacteriosis: Geographic distribution and recent advances in molecular diagnostic techniques. *Reprod. Domest. Anim.* 45 e221–e230. 10.1111/j.1439-0531.2009.01546.x 19929895

[B17] Naze F. Desvars A. Picardeau M. Bourhy P. Michault A. (2015). Use of a new high resolution melting method for genotyping pathogenic *Leptospira* spp. *PLoS One* 10:e0127430. 10.1371/journal.pone.0127430 26154161 PMC4496072

[B18] OIE (2021). *Manual of diagnostic tests and vaccines for terrestrial animals 2021.* Paris: Office international des épizootie.

[B19] On S. L. Holmes B. (1991a). Reproducibility of tolerance tests that are useful in the identification of campylobacteria. *J. Clin. Microbiol.* 29, 1785–1788. 10.1128/JCM.29.9.17851788.1991 1774297 PMC270211

[B20] On S.L. Holmes B. (1991b). Effect of inoculum size on the phenotypic characterization of Campylobacter species. *J. Clin. Microbiol.* 29, 923–926. 10.1128/JCM.29.5.923-926.1991 2056060 PMC269909

[B21] Pakbin B. Basti A. A. Khanjari A. Brück W. M. Azimi L. Karimi A. (2022). Development of high-resolution melting (HRM) assay to differentiate the species of *Shigella* isolates from stool and food samples. *Sci. Rep.* 12 1–13. 10.1038/s41598-021-04484-1 35013489 PMC8748861

[B22] Polo C. García-Seco T. Hernández M. Fernández V. Rodríguez-Lázaro D. Goyache J. (2021). Evaluation of PCR assays for *Campylobacter fetus* detection and discrimination between C. Fetus subspecies in bovine preputial wash samples. *Theriogenology* 172 300–306. 10.1016/j.theriogenology.2021.06.020 34311221

[B23] Silva M. F. Duarte A. Pereira G. Mateus L. Lopes-da-Costa L. Silva E. (2020a). Assessment of *Campylobacter fetus* subsp. Venerealis molecular diagnosis using clinical samples of bulls. *BMC Vet. Res.* 16:410. 10.1186/s12917-020-02634-7 33121492 PMC7596931

[B24] Silva M. F. Gonçalo P. Carneiro C. Hemphill A. Mateus L. Lopes-da-Costa L. (2020b). *Campylobacter portucalensis* sp. nov., a new species of *Campylobacter* isolated from the preputial mucosa of bulls. *PLoS One* 15:e0227500. 10.1371/journal.pone.0227500 31923228 PMC6953823

[B25] Silveira C. da S. Fraga M. Giannitti F. Macías-Rioseco M. Riet-Correa F. (2018). Diagnosis of bovine genital campylobacteriosis in South America. *Front. Vet. Sci.* 5:321. 10.3389/fvets.2018.00321 30619902 PMC6302017

[B26] Spence R. P. Bruce I. R. McFadden A. M. J. Hill F. I. Tisdall D. Humphrey S. (2011). Short communications: Cross-reaction of a *Campylobacter fetus* subspecies venerealis real-time PCR. *Vet. Rec.* 168:131. 10.1136/vr.c5264 21257600

[B27] Sprenger H. Zechner E. L. Gorkiewicz G. (2012). So close and yet so far — molecular microbiology of *Campylobacter fetus* subspecies. *Eur. J. Microbiol. Immunol.* 2 66–75. 10.1556/eujmi.2.2012.1.10 24611123 PMC3933992

[B28] Untergasser A. Cutcutache I. Koressaar T. Ye J. Faircloth B. C. Remm M. (2012). Primer3-new capabilities and interfaces. *Nucleic Acids Res.* 40 1–12. 10.1093/nar/gks596 22730293 PMC3424584

[B29] Van Bergen M. A. P. Dingle K. E. Maiden M. C. J. Newell D. G. Van Der Graaf-Van Bloois L. Van Putten J. P. M. (2005). Clonal nature of *Campylobacter fetus* as defined by multilocus sequence typing. *J. Clin. Microbiol.* 43 5888–5898. 10.1128/JCM.43.12.5888-5898.2005 16333072 PMC1317208

[B30] van der Graaf-van Bloois L. Duim B. Miller W. G. Forbes K. J. Wagenaar J. A. Zomer A. (2016a). Whole genome sequence analysis indicates recent diversification of mammal-associated *Campylobacter fetus* and implicates a genetic factor associated with H2S production. *BMC Genomics* 17:713. 10.1186/s12864-016-3058-7 27599479 PMC5013579

[B31] van der Graaf-van Bloois L. Miller W. G. Yee E. Gorkiewicz G. Forbes K. J. Zomer A. L. (2016b). *Campylobacter fetus* subspecies contain conserved type IV secretion systems on multiple genomic islands and plasmids. *PLoS One* 11:e0152832. 10.1371/journal.pone.0152832 27049518 PMC4822827

[B32] van der Graaf-Van Bloois L. Miller W. G. Yee E. Rijnsburger M. Wagenaar J. A. Duim B. (2014). Inconsistency of phenotypic and genomic characteristics of *Campylobacter fetus* subspecies requires reevaluation of current diagnostics. *J. Clin. Microbiol.* 52 4183–4188. 10.1128/JCM.01837-14 25232170 PMC4313284

[B33] van der Graaf-van Bloois L. van Bergen M. A. P. van der Wal F. J. de Boer A. G. Duim B. Schmidt T. (2013). Evaluation of molecular assays for identification *Campylobacter fetus* species and subspecies and development of a C. Fetus specific real-time PCR assay. *J. Microbiol. Methods* 95 93–97. 10.1016/j.mimet.2013.06.005 23810970

[B34] Wagenaar J. A. Van Bergen M. A. P. Newell D. G. Grogono-Thomas R. Duim B. (2001). Comparative study using amplified fragment length polymorphism fingerprinting, PCR genotyping, and phenotyping to differentiate *Campylobacter fetus* strains isolated from animals. *J. Clin. Microbiol.* 39 2283–2286. 10.1128/JCM.39.6.2283-2286.2001 11376071 PMC88125

[B35] Ye J. Coulouris G. Zaretskaya I. Cutcutache I. Rozen S. Madden T. L. (2012). Primer-BLAST: A tool to design target-specific primers for polymerase chain reaction. *BMC Bioinformatics* 13:134. 10.1186/1471-2105-13-134 22708584 PMC3412702

[B36] Zhang J. Peng, Liu Z. Cheng, Jiang J. Xiu (2021). Rapid detection of *Mycoplasma mycoides* subsp. Capri and *Mycoplasma capricolum* subsp. Capripneumoniae using high-resolution melting curve analysis. *Sci. Rep.* 11 1–8. 10.1038/s41598-021-93981-4 34321522 PMC8319336

